# Mr. Franks, on Variolous Contagion

**Published:** 1800-12

**Authors:** John Franks

**Affiliations:** Smith Street, Westminster


### *To the Editors of the Medical and Phyſical Journal.*

Gentlemen,

I Have read with pleaſure in your uſeful Journal the various proofs which have been adduced in ſupport of the benefits likely to acrue to the community by the introduction of vaccine inoculation.

But, although willing to allow the new practice the merit of of inducing a more mild difeafe than that which variolous inoculation, with all its improvements, can *always* promife; yet, it occurs to me, that the fuperiority of vaccine over variolous inoculation, is fomewhat over-rated, when *it alone* is afferted to be incapable of producing infection, except by actual contact.

This fuperiority will not, perhaps, on a ftrict examination, be found to obtain. The common prejudice, that variolous inoculation gives origin to a contagious difeafe, or is itfelf contagious, it may not be impoffible to remove.

The improved variolous inoculation would have been more extenfively ufeful, had it not been for this objection, which has been propagated with fome other objections, which it has furvived, by medical perfons of this country and century.

The other objections to the practice of inoculating fmall-pox have, in a great meafure, been long fince abandoned by men of the fame clafs with thofe with whom they originated,—I allude to the ideas of the danger to be apprehended from a double infection; the great danger of communicating with the variolous matter, fcrofulous and other difeafes; and the infecurity which it has been faid the practice afforded; inftances having every now and then, during its infancy, been related, of perfons having natural fmall-pox after a previous inoculation.

Thefe objections, I fay, to variolous inoculation, are given up by profeffional men; but the other, the contagious nature of the difeafe induced by this inoculation, is ftill entertained by many men of eminence in the profeffion.

It would have been fortunate for mankind, and particularly fo for the inhabitants of the cities of London and Weftminfter, and the places adjacent, if the poor multitude would have parted with their prejudices, relative to variolous inoculation, and which they had imbibed from their fuperiors in knowledge, at the time when the latter could have wifhed: But they have neither been fo complaifant to others, or fo friendly to themfelves.

Practitioners who are refident in the above places, know very well, that when fmall-pox is in a houfe where there are many children and adults liable to the difeafe, the propofal to inoculate, gratuitoufly, all thofe who are not exempt, is too often difregarded by themfelves or relations.

It is in vain that we expoftulate in thefe fituations, and endeavour to convince them of the non-exiftence of a double infection, or of an accumulation of difeafe; for the contrary opinion is too firmly impreffed to be eafily obliterated.

Were it not on account of this unhappy perfuafion, which, it would feem, cannot be parted with by the loweft clafs of people, together with the no lefs unfortunate one, exifting ftill in the minds of well-informed men, namely, that the variolous inoculation is contagious, the Bills of Mortality would exhibit a far lefs number of deaths from fmall-pox than they have heretofore done; inasmuch as in ninety-nine inftances in every hundred, we fhould be able to anticipate the cafual fmall-pox, which probably would prove fatal in a great number of cafes, by the production of a difeafe fo mild in its operation, as in a general way to occafion little or no inconvenience under a judicious mode of treatment.

But experience has proved, that fometimes, and under the moft judicious management, an adverfe cafe will prefent. Vaccine inoculation is recommended to the public as a fubftitute, not, as I apprehend becaufe no adverfe cafe has ever occurred, or will ever occur, but, becaufe the number of thefe is likely, from our prefent experience, to be confiderably lefs than from the variolous.

Now, as few phyficians would affert pofitively, that no unfavourable event can poffibly happen from an employment of the fubftitute, parents may be found who poffibly may obferve, we are not anxious to have an unneceffary difeafe, obtruded on our progeny to which they are not obnoxious; as from the evidence which we have of the fafety of the variolous inoculation, we fhall prefer the human to the vaccine difeafe. That the fubftitution of a difeafe of brute origin will meet with oppofition from fome perfons is apparent; for Dr. Waterhoufe finds it neceffary in America to change the name of it. The Doctor has obferved, that people revolt at the term Cow-pox.*

I fhall not, I imagine, be fufpected by any of your readers, of an intention to create prejudices in the minds of the common people againft the new inoculation, while I make my obfervations on it, through the medium of a vehicle with which they are unacquainted. If this inoculation be readily accepted by the public, as a fubftitute for the former, or with the view of exemption from cafual fmall-pox, it will meet with a more favourable reception than the other has done; and a favourable reception it ought to meet with, or the fanguine expectations of fome writers on the fubject will never be realifed.

I have faid that the fuperiority of vaccine over variolous inoculation is fomewhat over-rated, when it is afferted, that it

---

* See Med. and Phys. Journal, No. XXI. p. 471.

*it alone* is incapable of communicating infection through the medium of the atmosphere.

"The small-pox are chiefly destructive to the poor; the rich, for the most part, secure their children from the casual small-pox by inoculation; but this is done at the risk of their less intelligent neighbours. Thus an operation which confers an invaluable blessing on the few intelligent individuals who adopt it, becomes injurious to the less intelligent part of the community. Humanity calls aloud on us to substitute a disease that brings security to those who seek it, and spreads danger to none."†

Thus is the character of the one inoculation magnified at the expence of the other. It would be taking up too large a portion of your publication at present, to prosecute the intended enquiry, whether this charge which has been made against the variolous inoculation, be well or ill founded. But should you be of opinion that these remarks are not altogether irrelevant, and give them a place in the next Number, I will endeavour to send you a communication for a future one; to evince that the above charge is without foundation. I am,

Gentlemen,

Your obedient humble servant,

JOHN FRANKS.

*Smith Street, Westminster,*

*November* 7, 1800.

† Vide Dr. Cappe on the Cow-pox, No. XXI. p. 438.

